# Editorial: Gastronomic Science

**DOI:** 10.3389/fphys.2020.612921

**Published:** 2020-10-27

**Authors:** Suzanne Devkota, Leon G. Fine

**Affiliations:** ^1^Cedars Sinai Medical Center, Los Angeles, CA, United States; ^2^Karsh Division of Gastroenterology and Hepatology, Los Angeles, CA, United States; ^3^Department of Medicine, Los Angeles, CA, United States

**Keywords:** gastronomy, taste, smell, microbiota, cooking

In this Frontiers Special Research Topic on “Gastronomic Sciences,” we feature three review commentaries that explore the history and evolution of gastronomy, both as a philosophy and an art that has influenced human cooking and feeding behavior, and two original research studies involving taste perception.

Two separate reviews discuss “disruptors” in gastronomic sciences that fundamentally changed perceptions of food structure, function, and access: del Moral applies Thomas Kuhn's “The Structure of Scientific Revolutions,” to the changing paradigms of gastronomy in Gastronomic Paradigms in Contemporary Western Cuisine: From French Haute Cuisine to Mass Media Gastronomy and Fine explains how La Varenne's now famous cookbook, Le Cuisinier Francois, for the first time made French cuisine accessible to the home cook in The Transformative Influence of La Varenne's Le Cuisinier Francois (1651) on French Culinary Practice. These insights address the evolution of gastronomy and how the most influential works are ultimately the ones that reach the most people. This concept takes cooking from the elitist realm of chefs and restaurants into the home which David Zilber, former head of fermentation at Noma, has referred to as “democratizing deliciousness.”

In the third review, Sense of smell as the central driver of Pavlovian appetite behavior in mammals, Fine and Riera, revisit Pavlov's sensory conditioning experiments whereby digestive secretions could be stimulated in the absence of ingestion of food. Pavlov's findings imparted the power of the brain to control digestion, but Pavlov did not attribute any one sense- sight, smell, sound etc. as the driving stimulus. Here, Fine and Riera posit that the sense of smell may well be the strongest driver of food perception in mammals and probably in man.

In an original research contribution, Pan et al. explore the fascinating phenomenon of high-altitude associated appetite loss in Association between single nucleotide polymorphisms in PPARA and EPAS1 genes and high-altitude appetite loss in Chinese young men. High altitude is known to cause unexplained shifts in not only appetite but also taste. In this report, examine a cohort of 416 Chinese men traveling by airplane from Chengdu to Lhasa, experiencing rapid ascension to and elevation of 3,700 m, and identify genetic polymorphisms associated with those individuals experiencing appetite-loss.

Finally, Poteres et al. report the effects of diet and antibiotics on the small bowel microbiota in mice (Selective regional alteration of the gut microbiota by diet and antibiotics). The small bowel has been underrepresented in microbiome research due to large reliance on stool samples for many microbiome studies, which are not necessarily representative of microbial communities in the proximal gut. Here the authors specifically focus on post-mortem collection of small bowel mucosa, and, interestingly, find that while antibiotics alter the microbiota throughout the small intestines, diet only appear to affect the community composition of microbiota in the jejunum and cecum.

## Author Contributions

All authors listed have made a substantial, direct and intellectual contribution to the work, and approved it for publication.

## Conflict of Interest

The authors declare that the research was conducted in the absence of any commercial or financial relationships that could be construed as a potential conflict of interest.

